# The impact of high‐intensity interval training on ventricular remodeling in patients with a recent acute myocardial infarction—A randomized training intervention pilot study

**DOI:** 10.1002/clc.23277

**Published:** 2019-10-10

**Authors:** Lukas‐Daniel Trachsel, Louis‐Philippe David, Mathieu Gayda, Christine Henri, Douglas Hayami, Nathalie Thorin‐Trescases, Éric Thorin, Mélissa‐Anne Blain, Mariève Cossette, Julie Lalongé, Martin Juneau, Anil Nigam

**Affiliations:** ^1^ Cardiovascular Prevention and Rehabilitation (ÉPIC) Center Montreal Heart Institute, Université de Montréal Montreal Quebec Canada; ^2^ Department of Medicine, Faculty of Medicine Université de Montréal Montreal Quebec Canada; ^3^ University Clinic for Cardiology, Inselspital, Bern University Hospital University of Bern Bern Switzerland; ^4^ Research Center Montreal Heart Institute, Université de Montréal Montreal Quebec Canada; ^5^ Department of Surgery, Faculty of Medicine Université de Montréal Montreal Quebec Canada; ^6^ Montreal Health Innovations Coordinating Center Montreal Heart Institute Montreal Quebec Canada

**Keywords:** aerobic exercise, cardiac remodeling, coronary heart disease, interval training secondary prevention

## Abstract

**Background:**

Aerobic exercise training is associated with beneficial ventricular remodeling and an improvement in cardiac biomarkers in chronic stable heart failure. High‐intensity interval training (HIIT) is a time‐efficient method to improve $\dot{V}O_{2\text{peak}}$ in stable coronary heart disease patients. This pilot study aimed to compare the effect of HIIT on ventricular remodeling in patients with a recent acute myocardial infarction (AMI).

**Methods:**

Nineteen post‐AMI patients were randomized to either HIIT (n = 9) or usual care (n = 10). A cardiopulmonary exercise test (CPET), transthoracic echocardiography, and cardiac biomarker assessment (ie, N‐terminal pro B‐type natriuretic peptide levels and G protein‐coupled receptor kinase 2 expression) were performed before and after a 12‐week training intervention. CPET parameters including oxygen uptake efficiency slope (OUES) and $\dot{V}O_{2}$ at the first ventilatory threshold ($\dot{V}O_{2}$ VT1) were calculated. left ventricular (LV) structural and functional echocardiographic parameters including myocardial strain imaging were assessed.

**Results:**

$\dot{V}O_{2\text{peak}}$ and OUES improved solely in the HIIT group (*P* < .05 for group/time, respectively). There was a significant training effect for the improvement of peak work load in both groups (*P* < .05). O_2_ pulse and $\dot{V}O_{2}$ at VT1 both improved only in the HIIT group (*P* < .05 for time, no interaction). HIIT improved radial strain and pulsed‐wave tissue Doppler imaging derived e′ (*P* < .05 for time, no interaction). Cardiac biomarkers did not change in either group.

**Conclusions:**

In post‐AMI patients, HIIT lead to significant improvements in prognostic CPET parameters compared to usual care. HIIT was associated with favorable ventricular remodeling regarding certain echocardiographic parameters of LV function.

## 1 INTRODUCTION

Acute myocardial infarction (AMI) can induce changes in left ventricular (LV) topography (ie, ventricular remodeling) and is a major contributor in the development of heart failure despite advances in coronary revascularization and optimal medical therapy.^1^ Myocardial strain imaging using speckle‐tracking echocardiography allows quantification of regional and global LV function and has been increasingly implemented in clinical practice.^2^ It is more sensitive for the detection of subclinical LV changes as compared to standard LV ejection fraction (LVEF) measurement.^3^ The best evaluated parameter, global longitudinal strain (GLS) is superior to LVEF in the prediction of prognosis and cardiac remodeling after AMI.^4^, ^5^, ^6^ Among others, cardiac β‐adrenergic receptor (β‐AR) signal dysregulation represents a hallmark abnormality potentially leading to LV remodeling post‐AMI and progression to heart failure. β‐AR kinase (GRK2) is the most abundant G protein‐coupled receptor kinase expressed in the heart.^7^ Importantly, abnormalities of β‐AR signaling in the failing heart, including GRK2 over‐expression, are mirrored in circulating white blood cells (ie, lymphocytes) and correlate with severity of LV dysfunction.^8^ Therefore, GRK2 provides potential as a biomarker of cardiac dysfunction.^9^

Exercise‐based secondary prevention programs have confirmed improvements in mortality and morbidity in patients with stable coronary heart disease (CHD) and after AMI, respectively.^10^, ^11^ The importance of starting aerobic exercise training early post‐AMI and the beneficial effects on LV remodeling have been emphasized in a recent meta‐analysis.^12^ Furthermore, aerobic exercise training has been shown to be associated with a lowering of GRK2 expression and to predict outcomes in patients with chronic ischemic heart failure in a prospective study.^13^ High‐intensity interval training (HIIT) is more effective at improving $\dot{V}O_{2\text{peak}}$ and can be performed safely compared to the more established moderate‐intensity continuous exercise training (MICET) in stable CHD patients.^14^, ^15^, ^16^

However, most prior studies included predominantly stable patients. To the best of our knowledge, the effect of HIIT on cardiac remodeling including advanced echocardiography (ie, myocardial strain imaging) and GRK2 expression has not yet been studied in patients with a recent AMI. This pilot investigation aimed to evaluate the effect of HIIT on cardiopulmonary exercise test (CPET) variables, left ventricular remodeling, and GRK2 expression in CHD patients who recently suffered an AMI. We hypothesized that HIIT would result in a higher $\dot{V}O_{2\text{peak}}$ improvement and a more favorable cardiac remodeling with a substantial reduction in GRK2 expression compared to a usual care group.

## 2 MATERIALS AND METHODS

### 2.1 Participants

Subjects with an AMI within the preceding 6 weeks referred for cardiac rehabilitation at the Cardiovascular Prevention and Rehabilitation (ÉPIC) Center of the Montreal Heart Institute were enrolled in a longitudinal, randomized prospective clinical training intervention study. They had access to multidisciplinary educational services usually offered in a secondary prevention program (ie, smoking cessation, nutritional counseling, etc.). Details on the inclusion and exclusion criteria have been previously described elsewhere.^17^, ^18^ Importantly, AMI (ST elevation myocardial infarction or non‐ST elevation myocardial infarction) was based on the universal definition.^19^ For more detailed information on the exclusion criteria see section S1 of the Supporting Information. Patients had to be stable with regard to symptoms and doses of medication during the 4 weeks prior to enrolment.

Although by definition the study was unblinded, individuals involved in data assessment and analysis were blinded to the allocation group (assessor‐blinded study). The study protocol was approved by the Research Ethics and New Technology Development Committee (CERDNT) of the Montreal Heart Institute (http://ClinicalTrials.gov identifier number: NCT02048696).

### 2.2 Study design and measurement

Baseline clinical assessment (ie, medical history, physical examination, and anthropometric measurements), blood analysis, transthoracic echocardiography, and CPET were performed at baseline and after completion of the program (for more detailed information on study design and measurement see section S2 of the Supporting Information).

### 2.3 Maximal CPET

Maximal CPET was performed on a cycle ergometer (Ergoline 800S, Bitz, Germany) according to the recommendations of the American Heart Association, and as previously published (for more detailed information see section S3 of the Supporting Information).^20^, ^21^, ^22^

### 2.4 Transthoracic echocardiography

Standard transthoracic 2D echocardiography was performed on a Vivid 9 cardiac ultrasound system with a 7.5‐MHz transducer (GE Medical system, New Jersey). All echocardiographic images were obtained by two cardiology fellows using standard tomographic views. All data were stored on an external hard‐drive and analyzed offline on a commercially available workstation (EchoPAC, GE Healthcare) by a cardiology fellow and checked by single experienced cardiologist with several years of expertise. Traditional echocardiographic parameters of LV dimension, and systolic and diastolic function were assessed based on the most recent recommendations.^23^, ^24^

### 2.5 2D speckle‐tracking strain analysis

Peak systolic LV longitudinal strain and strain rates were assessed using standard 2D apical four‐chamber, two‐chamber, and three‐chamber view using speckle‐tracking analysis.^25^ All images were recorded using high frame rate loops (50‐80 Hz) for reliable analysis by the software. Manual tracing of the endocardial borders on an end‐systolic frame (aortic valve closure) was performed and the myocardial region of interest was adjusted to include all the endocardium and epicardium, excluding the pericardium. Automatically tracing was then applied on subsequent frames. Adequate tracing for each segment was verified and manually corrected, if necessary. If tracing was still judged incorrect, the specific segment was excluded from the global strain measurement. If more than two segments were discarded, GLS and strain rates were not reported for that patient. The GLS and strain rates were determined by averaging all values of the 18 segments of the three views. Strain analysis with optimal tracking was feasible in 93% of all segments.

### 2.6 Blood samples and biomarkers

Blood samples were obtained by venipuncture in the antecubital vein for the evaluation of N‐terminal pro B‐type natriuretic peptide (NT‐pro BNP) and GRK2. The blood was then centrifuged to separate the cellular and plasmatic fraction and was stored at −80°C until the day of the assay. N‐terminal pro B‐type natriuretic peptide was assessed by electrochemiluminescence immunoassay on an Elecsys 2010 analyzer using Roche assay kits (Roche Diagnostic, Mannheim, Germany) according to the manufacturer's instructions.

### 2.7 G protein‐coupled receptor kinase 2

The expression of G protein‐coupled receptor kinase 2 (GRK2) was measured by Western blotting (for more detailed information on GRK2 evaluation see section S4 of the Supporting Information).

### 2.8 Exercise training intervention

Patients were randomized to either a 12‐week structured exercise training program including two weekly supervised HIIT sessions or a usual care group. An additional resistance training (RT) was performed following each HIIT session. All trainings were center‐based under supervision of an experienced kinesiologist. The HIIT training protocol was recently described by Guiraud et al.^21^ Following a 5‐minute warm‐up at 30% of peak work load obtained at the CPET, patients performed two to three sets of 6 to 8 minutes with repeated bouts of 15 to 30 seconds at 100% of peak work load alternated by 15 to 30 seconds of passive recovery. The targeted Borg rating of perceived exertion (RPE) was set at 15 during the HIIT bouts. The sets were separated by a 5‐minute active recovery phase at 30% of peak work load. The training session was terminated by a 5‐minute cool‐down phase at 30% of peak work load.^26^ RT consisted of 20 minutes of circuit weight training performed with elastic bands and free weight adapted to each patient's capacity. For each muscle group, patients performed one set of 15 to 20 repetitions, followed by a 30‐second rest period at a target RPE of 15.^26^

### 2.9 Usual care group

The control group received recommendations regarding physical activity for a period of 12 weeks by their discharging cardiologist. If there were no recommendations at discharge, physical activity recommendations consistent with recent guidelines were given. Patients were encouraged for 30 to 60 minutes of moderate‐intensity (target RPE of 12‐14) at least 5 days and preferably 7 days per week.^27^ Following completion of the study, subjects randomized into the usual care group had the opportunity to participate in structured supervised exercise training program offered by the ÉPIC Center of the Montreal Heart Institute.

### 2.10 Statistical analyses

Data are presented as mean ± SD for continuous variables, while frequencies and percentages are presented for categorical variables. Baseline characteristics were compared between the two groups using Student *t* test in case of continuous variables and categorical variables were compared using chi‐square test. Repeated measures ANOVA models were used to study the CPET and echocardiographic parameters across time and between groups. Models with time, group, and group × time interaction as independent variables were used. The group × time interaction was the main focus of the analysis as it tested the difference in the change (post‐pre) between the two groups. As a measure of effect size to evaluate the strength of the intervention effect (HIIT) vs usual care, the Hedge's g calculated by the formula below was presented.

Hedge's g = *M*_1_ − *M*_2_/sqrt[((n_1_ − 1)SD_1_^2^ + (n_2_ − 1)SD_2_^2^)/(n_1_ + n_2_ − 1)].where *M*_1_ = mean of the change (post‐pre) in HIIT group, *M*_2_ = mean of the change (post‐pre) in usual care group, SD_1_ = SD of the change (post‐pre) in HIIT group, SD_2_ = SD of the change (post‐pre) in usual care group, n_1_ = number of subjects in HIIT group, n_2_ = number of subjects in usual care group.

An absolute value between 0.5 and 0.8 for g was considered as a medium effect and an absolute value >0.8 for g was considered as a high effect. In addition, under the repeated measures ANOVA model, the change (post‐pre) within each group was formally tested against zero. All analyses were done with SAS version 9.4 (SAS Institute Inc., Cary, North Carolina) and conducted at the 0.05 significance level.

## 3 RESULTS

### 3.1 Clinical characteristics

A total of 19 were included in the final analysis (HIIT: n = 9, usual care group: n = 10). Baseline clinical characteristics of CHD patients with a recent AMI, either randomized to the HIIT or usual care group are summarized in Table 1. There were no significant differences between the groups with regards to demographic data, event details and baseline medication except for a lower number of patients on inhibitors of the renin angiotensin aldosterone system (ACE inhibitor or angiotensin receptor blocker) in the HIIT group (*P* < .05).

**Table 1:** Baseline characteristics of CHD patients randomized to the HIIT or usual care group

Variable	HIIT, n = 9 (mean ± SD)	Usual care, n = 10 (mean ± SD)	*P*‐value
Age (y)	60 ± 10	57 ± 13	.494
Male sex	6 (67)	7 (70)	.876
Height (m)	1.70 ± 0.13	1.71 ± 0.11	.798
Weight (kg)	81.9 ± 9.1	86.5 ± 18.2	.508
Body mass index (kg/m^2^)	28.7 ± 4.2	29.4 ± 4.8	.719
Lean body mass	58.3 ± 10.8	58.6 ± 13.8	.956
Fat mass (%)	29.0 ± 9.6	32.3 ± 8.3	.433
Systolic BP (mm Hg)	116 ± 11	121 ± 10	.300
Diastolic BP (mm Hg)	68 ± 9	74 ± 10	.184
*Event characteristics*			
AMI	9 (100)	10 (100)	NA
STEMI	4 (44)	8 (80)	0.109
Primary PCI	9 (100)	10 (100)	NA
*Cardiovascular risk profile*			
Active smoking	1 (11)	2 (20)	.596
Hypertension	5 (56)	5 (50)	.809
Type 2 diabetes mellitus	0 (0)	1 (10)	.330
Dyslipidemia	7 (78)	10 (100)	.115
*Baseline medication*			
Aspirin	8 (89)	10 (100)	.279
DAPT	9 (100)	10 (100)	NA
Lipid‐lowering therapy	9 (100)	10 (100)	NA
RAAS inhibitors	2 (22)	7 (70)	.037
Beta‐blockers	7 (78)	9 (90)	.466
CCB	1 (11)	1 (10)	.937

Note: Data are expressed as mean ± SD, dichotomous variables are expressed as numbers and percentages.

Abbreviations: AMI, acute myocardial infarction; BP, blood pressure; CCB, calcium channel blocker; CHD, coronary heart disease; DAPT, dual antiplatelet therapy; HIIT, high‐intensity interval training; NA, not applicable; PCI, percutaneous coronary intervention; RAAS inhibitors; inhibitors of the renin angiotensin aldosterone system; STEMI, ST elevation myocardial infarction.

### 3.2 Maximal CPET parameters

Table 2 shows maximal CPET parameters pre‐ and post‐training in CHD patients randomized to the HIIT or usual care group (for a more detailed description of the results of CPET parameters we refer to section S5 of the Supporting Information).

**Table 2:** CPET parameters pre‐ and post‐training in CHD patients randomized to the HIIT or usual care group

		HIIT, n = 9 (mean ± SD)	Usual care, n = 10 (mean ± SD)	Group × time interaction *P*‐value (Hedge's g)
$\dot{V}O_{2}$ peak/LBM (mL/min/kg)	Pre	27.6 ± 6.9	29.2 ± 4.3	0.012 (1.29)
	Post	30.6 ± 6.6	29.3 ± 4.6	
	Δ (post‐pre)	3.1 ± 2.4	0.1 ± 2.3	
	*P*‐value Δ (post‐pre)*	0.0009	0.879	
$\dot{V}O_{2}$ peak % predicted	Pre	93.4 ± 27.0	90.9 ± 26.4	0.026 (1.12)
	Post	101.4 ± 29.8	90.0 ± 24.7	
	Δ (post‐pre)	8.0 ± 7.8	−0.9 ± 8.1	
	*P*‐value Δ (post‐pre)*	0.008	0.725	
Peak work load (W)	Pre	120.0 ± 46.3	127.1 ± 39.9	0.533 (0.29)
	Post	132.2 ± 49.6	135.7 ± 43.5	
	Δ (post‐pre)	12.2 ± 12.8	8.6 ± 12.0	
	*P*‐value Δ (post‐pre)*	0.009	0.042	
Peak RER	Pre	1.19 ± 0.05	1.16 ± 0.08	0.182 (−0.78)
	Post	1.14 ± 0.09	1.14 ± 0.05	
	Δ (post‐pre)	−0.05 ± 0.06	−0.00 ± 0.06	
	*P*‐value Δ (post‐pre)*	0.034	0.723	
RPP	Pre	22 205 ± 4581	24 361 ± 5802	0.215 (0.59)
	Post	22 430 ± 4509	22 689 ± 4635	
	Δ (post‐pre)	224 ± 3964	−1672 ± 2328	
	*P*‐value Δ (post‐pre)*	0.836	0.117	
Peak sBP (mm Hg)	Pre	178.0 ± 26.2	185.1 ± 25.6	0.068 (0.90)
	Post	179.9 ± 23.6	175.2 ± 21.7	
	Δ (post‐pre)	1.9 ± 14.0	−9.9 ± 12.3	
	P‐value Δ (post‐pre)*	0.672	0.029	
Peak dBP (mm Hg)	Pre	78.3 ± 11.7	79.0 ± 10.2	0.882 (0.07)
	Post	74.7 ± 10.1	76.0 ± 10.2	
	Δ (post‐pre)	−3.7 ± 7.1	−3.0 ± 11.3	
	*P*‐value Δ (post‐pre)*	0.268	0.337	
Peak HR (bpm)	Pre	124.3 ± 13.8	130.6 ± 21.2	0.799 (0.12)
	Post	124.8 ± 22.3	129.5 ± 22.5	
	Δ (post‐pre)	0.4 ± 16.6	−1.1 ± 8.6	
	*P*‐value Δ (post‐pre)*	0.920	0.793	
HR Res (bpm)	Pre	59.6 ± 10.8	59.5 ± 20.3	0.958 (−0.03)
	Post	61.1 ± 14.5	61.2 ± 19.3	
	Δ (post‐pre)	1.5 ± 13.3	1.8 ± 7.3	
	*P*‐value Δ (post‐pre)*	0.745	0.466	
OUES	Pre	1619 ± 409	1832 ± 399	0.032 (1.08)
	Post	1830 ± 481	1838 ± 507	
	Δ (post‐pre)	211 ± 168	6 ± 209	
	*P*‐value Δ (post‐pre)*	0.004	0.918	
$\dot{V}E$/$\dot{\mathrm{VC}}O_{2}$ slope	Pre	32.4 ± 3.4	30.8 ± 4.7	0.358 (−0.43)
	Post	31.2 ± 3.1	30.6 ± 3.3	
	Δ (post‐pre)	−1.2 ± 1.7	−0.2 ± 3.0	
	*P*‐value Δ (post‐pre)*	0.157	0.852	
Δ$\dot{V}O_{2}$/ΔWork load slope	Pre	9.1 ± 1.7	10.5 ± 1.2	0.178 (0.68)
	Post	9.4 ± 1.1	10.0 ± 1.2	
	Δ (post‐pre)	0.3 ± 0.8	−0.5 ± 1.6	
	*P*‐value Δ (post‐pre)*	0.234	0.363	
O_2_ pulse (mL/beat)	Pre	13.5 ± 4.0	13.2 ± 2.6	0.110 (0.77)
	Post	15.1 ± 4.3	13.5 ± 3.3	
	Δ (post‐pre)	1.5 ± 1.4	0.3 ± 1.7	
	*P*‐value Δ (post‐pre)*	0.011	0.588	
$\dot{V}O_{2}$ at VT1 (%)	Pre	67 ± 24	64 ± 23	0.256 (0.15)
	Post	76 ± 23	67 ± 23	
	Δ (post‐pre)	8 ± 7	3 ± 12	
	*P*‐value Δ (post‐pre)*	0.023	0.373	

Note: Variables are expressed as means ± SD.

Abbreviations: BP, blood pressure; CHD, coronary heart disease; CPET, cardiopulmonary exercise test; HIIT, high‐intensity interval training; HR, heart rate; HR Res, heart rate reserve; OUES, oxygen uptake efficiency slope; RER, Respiratory exchange ratio; RPP, rate‐pressure product at peak exercise; $\dot{\mathrm{VE}}$/$\dot{V}{\mathrm{CO}}_{2}$ slope, ventilatory efficiency slope; $\dot{V}O_{2}$, oxygen consumption; VT1, first ventilatory threshold.

*P‐value Δ (post‐pre) within group.

### 3.3 Echocardiographic parameters

Echocardiographic parameters of LV geometry, systolic and diastolic function in both groups are summarized in Table 3 for a more detailed description of the results of echocardiographic parameters we refer to section S6 of the supplemental text).

**Table 3:** Echocardiographic parameters pre‐ and post‐training in CHD patients randomized to the HIIT or usual care group

		HIIT, n = 8 (mean ± SD)	Usual care, n = 10 (mean ± SD)	Group × time interaction *P*‐value (Hedge's g)
LVMI (g/m^2^)	Pre	80.8 ± 20.3	71.5 ± 16.7	0.147 (−0.72)
	Post	71.4 ± 17.5	75.5 ± 15.7	
	Δ (post‐pre)	−9.5 ± 21.8	4.0 ± 15.9	
	*P*‐value Δ (post‐pre)*	0.170	0.509	
LVEDVi (mL/m^2^)	Pre	52.5 ± 8.5	52.2 ± 15.6	0.574 (−0.44)
	Post	52.0 ± 14.4	54.3 ± 19.3	
	Δ (post‐pre)	−0.5 ± 15.1	6.0 ± 14.2	
	*P*‐value Δ (post‐pre)*	0.930	0.472	
LVEF (%)	Pre	65.9 ± 5.8	58.5 ± 8.5	0.114 (−0.90)
	Post	65.0 ± 7.9	60.8 ± 6.2	
	Δ (post‐pre)	−0.9 ± 5.6	3.6 ± 4.2	
	*P*‐value Δ (post‐pre)*	0.627	0.079	
GLS (%)	Pre	−20.5 ± 3.2	−18.1 ± 2.9	0.606 (0.33)
	Post	−21.7 ± 3.4	−19.9 ± 2.2	
	Δ (post‐pre)	−0.8 ± 3.2	−1.7 ± 2.6	
	*P*‐value Δ (post‐pre)*	0.383	0.076	
GLSR (s^−1^)	Pre	−0.95 ± 0.11	−0.97 ± 0.20	0.616 (0.06)
	Post	−1.07 ± 0.26	−1.14 ± 0.31	
	Δ (post‐pre)	−0.11 ± 0.28	−0.13 ± 0.21	
	*P*‐value Δ (post‐pre)*	0.298	0.042	
Circumferential strain (%)	Pre	−14.7 ± 2.1	−14.0 ± 5.7	0.967 (0.02)
	Post	−18.5 ± 5.3	−18.0 ± 5.0	
	Δ (post‐pre)	−4.3 ± 7.5	−4.5 ± 10.6	
	*P*‐value Δ (post‐pre)*	0.269	0.167	
Systolic SR (s^−1^)	Pre	−0.77 ± 0.11	−0.83 ± 0.33	0.634 (0.37)
	Post	−0.74 ± 0.87	−1.07 ± 0.35	
	Δ (post‐pre)	0.01 ± 0.99	−0.25 ± 0.50	
	*P*‐value Δ (post‐pre)*	0.981	0.193	
Radial strain (%)	Pre	28.8 ± 9.7	24.5 ± 6.1	0.450 (0.40)
	Post	41.6 ± 13.3	31.5 ± 12.2	
	Δ (post‐pre)	14.7 ± 10.8	9.4 ± 14.5	
	*P*‐value Δ (post‐pre)*	0.040	0.131	
Systolic SR (s^−1^)	Pre	1.15 ± 0.41	1.35 ± 0.84	0.476 (0.75)
	Post	1.75 ± 0.48	1.16 ± 0.46	
	Δ (post‐pre)	0.28 ± 0.28	−0.37 ± 1.04	
	*P*‐value Δ (post‐pre)*	0.549	0.621	
Peak E (cm/s)	Pre	68.5 ± 14.8	67.6 ± 17.7	0.699 (−0.19)
	Post	69.1 ± 11.0	71.7 ± 16.1	
	Δ (post‐pre)	0.6 ± 20.6	4.1 ± 15.6	
	*P*‐value Δ (post‐pre)*	0.933	0.428	
Peak A (cm/s)	Pre	71.9 ± 22.4	66.7 ± 16.0	0.214 (0.61)
	Post	77.8 ± 15.9	64.5 ± 20.8	
	Δ (post‐pre)	5.9 ± 13.6	−2.2 ± 12.8	
	*P*‐value Δ (post‐pre)*	0.224	0.604	
E/A ratio	Pre	1.02 ± 0.38	1.06 ± 0.38	0.140 (−0.74)
	Post	0.94 ± 0.36	1.21 ± 0.43	
	Δ (post‐pre)	−0.08 ± 0.33	0.15 ± 0.29	
	*P*‐value Δ (post‐pre)*	0.479	0.148	
TDI‐e′ septal (cm/s)	Pre	7.3 ± 1.2	8.3 ± 1.6	0.310 (0.50)
	Post	8.8 ± 1.4	8.9 ± 2.2	
	Δ (post‐pre)	1.5 ± 1.6	0.6 ± 2.0	
	*P*‐value Δ (post‐pre)*	0.032	0.310	
TDI‐e′ lateral (cm/s)	Pre	9.1 ± 3.1	9.7 ± 3.2	0.650 (0.21)
	Post	10.1 ± 2.2	10.1 ± 2.5	
	Δ (post‐pre)	1.0 ± 2.4	0.4 ± 3.0	
	*P*‐value Δ (post‐pre)*	0.317	0.650	
E/e′	Pre	8.7 ± 1.7	7.2 ± 1.7	0.103 (−0.82)
	Post	7.8 ± 2.0	8.4 ± 1.6	
	Δ (post‐pre)	−0.9 ± 2.4	1.2 ± 2.7	
	*P*‐value Δ (post‐pre)*	0.319	0.167	

*Note*: Variables are expressed as means ± SD.

Abbreviations: CHD, coronary heart disease; GLS, global longitudinal strain; GLSR, global longitudinal strain rate; HIIT, high‐intensity interval training; LVEF, left ventricular ejection fraction; LVEDVi, left ventricular end‐diastolic volume index; LVMI, left ventricular mass index; SR, strain rate; Peak A, peak late mitral inflow velocity; Peak E, peak early mitral inflow velocity; TDI‐e′ lateral, tissue Doppler imaging‐derived peak early diastolic lateral mitral annulus velocity; TDI‐e′ septal, tissue Doppler imaging‐derived peak early diastolic septal mitral annulus velocity; E/e′, peak early mitral inflow velocity to peak early diastolic mitral annulus velocity ratio.

*P‐value Δ (post‐pre) within group.

### 3.4 Cardiac biomarkers

**Figure 1:**
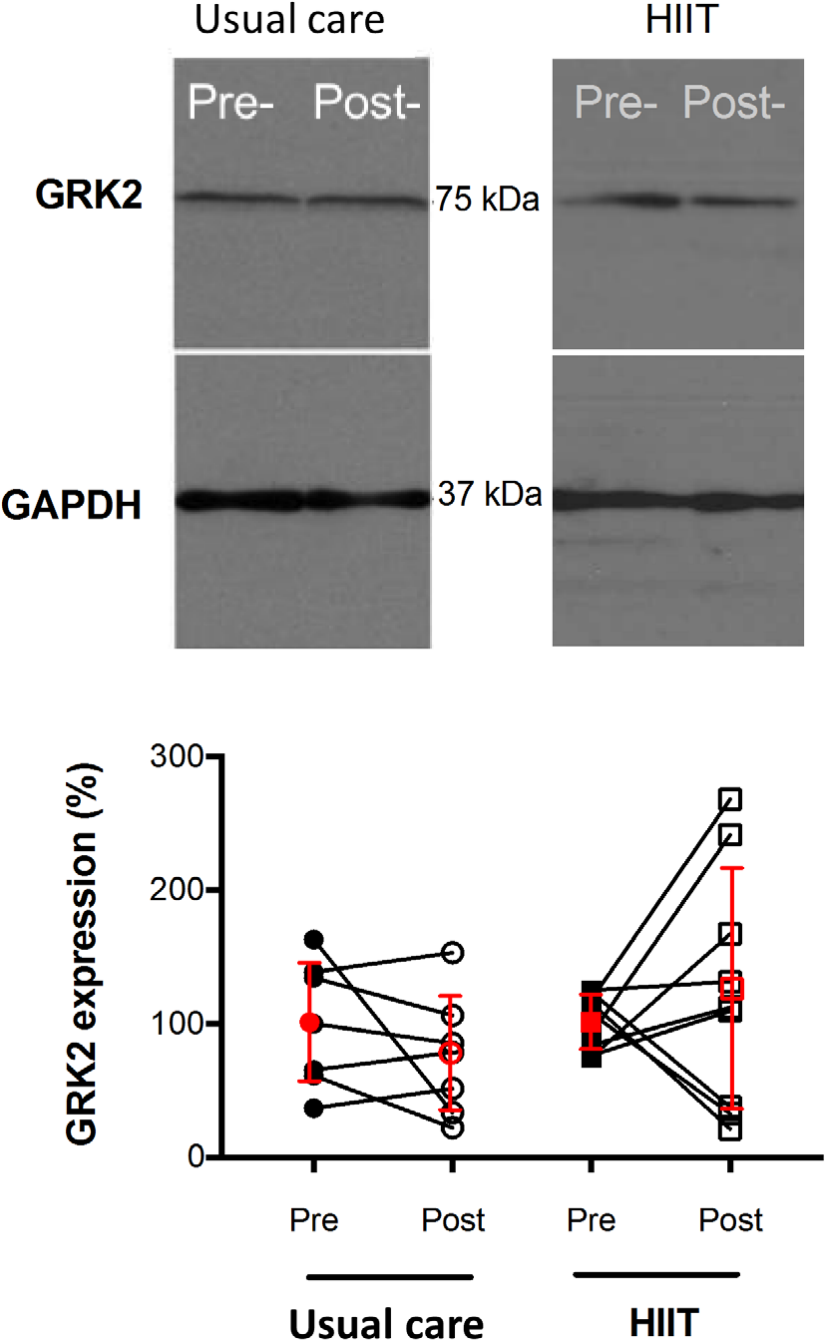
GRK2 protein expression pre‐ and post‐training with either usual care or HIIT. Top: typical Western blots are shown. Bottom: Paired individual GRK2 expressions (normalized to GAPDH and to 100% in pre‐training) are shown in each patient. In red, mean ± SD of the changes in GRK2 expression for each type of exercise is shown, n = 8 in MICET and n = 9 in HIIT. GRK2, G protein‐coupled receptor kinase 2; HIIT, high‐intensity interval training; MICET, moderate‐intensity continuous exercise training

N‐terminal pro B‐type natriuretic peptide levels were 290 ± 420 pg/mL in the HIIT and 200 ± 154 pg/mL in the usual care group at baseline. No significant group × time interaction was found (*P* = .647; *g* = −0.23) nor time effect but patients in the HIIT group exhibited a more pronounced decrease in NT‐pro BNP levels after completion of the training intervention. No significant group × time interaction was found for GRK2 expression (*P* = .128; *g* = −0.72) nor time effect. However, in the HIIT group, GRK2 expression decreased with training, while there was an increase in the usual care group (Δ post‐pre: −34.3 ± 54.3% in the HIIT and +23.0 ± 95.7% in the usual care group; *P* > .05 for Δ post‐pre in both groups). GRK2 protein expressions for the two groups pre‐ and post‐training are presented in Figure 1.

## 4 DISCUSSION

The main findings in our pilot investigation evaluating the effects of HIIT on ventricular remodeling in a highly vulnerable patient group (ie, patients who suffered a recent AMI) are: (a) $\dot{V}O_{2\text{peak}}$, oxygen uptake efficiency slope (OUES), and O_2_ pulse improved solely in the HIIT group. (b) HIIT overall exhibited a more favorable cardiac remodeling with regard to echocardiographic parameters of LV function as compared to the usual care group. (c) These changes were not associated with a significant change in cardiac biomarkers (ie, NT‐pro BNP levels and GRK2 expression).

### 4.1 The impact of HIIT on CPET parameters

Key CPET parameters like $\dot{V}O_{2\text{peak}}$, OUES, and O_2_ pulse (including O_2_ pulse trajectory) are highly relevant predictors of mortality and morbidity in CHD patients.^28^, ^29^, ^30^ Structured exercise training regardless of training modality has proven to improve these parameters.^29^, ^31^ However, in our study only patients in the HIIT group showed a substantial $\dot{V}O_{2\text{peak}}$ improvement and reached a normal age‐predicted $\dot{V}O_{2\text{peak}}$ after completion of the program with the identical level of exhaustion (ie, RER) as compared to the usual care group. This improvement is consistent with existing data in the identical population and with the biggest and latest of numerous meta‐analyses comparing HIIT and MICET in a population of CHD patients who reported the most pronounced improvements with HIIT after 7 to 12 weeks of training.^16^, ^32^ The finding that $\dot{V}O_{2}$ at the VT1 improved only in the HIIT group indicates an improvement in muscular function as a result of structured exercise training and is plausible.^33^

### 4.2 The impact of HIIT on echocardiographic parameters of cardiac remodeling

To the best of our knowledge, our pilot study for the first time shows beneficial effects of a HIIT program on cardiac remodeling in patients with a recent AMI. Existing literature has shown that aerobic exercise training starting early after AMI has no detrimental effects and even reverses ventricular remodeling in post‐AMI LV dysfunction.^12^, ^34^, ^35^ Additional data suggest a more favorable cardiac remodeling after HIIT as compared to the more established MICET or a control group in chronic stable post‐AMI patients with heart failure.^36^ However, the effects of HIIT in patients with a recent AMI and potential acute transient LV dysfunction have never been examined.

First of all, patients in our cohort were on optimal and stable medical therapy. The primary explanation for the lower number of patients on inhibitors of the renin angiotensin aldosterone system in the HIIT group is the lower number of STEMI patients in this group and the absence of LV dysfunction requiring this medication in the whole cohort before inclusion into the study. Importantly, after completion of the program all patients in our study except for one had a normal LVEF, left ventricular mass and volumes (indexed by body surface area), and no higher degree diastolic dysfunction (ie, diastolic dysfunction > grade I) based on recent recommendations.^23^, ^24^ In our pilot study, all these “conventional” parameters remained stable with HIIT, which is in line with other studies that employed lower training intensities^12^, ^35^ Furthermore, e′ septal as a parameter of diastolic function showed a significant improvement with training only in the HIIT group. However, there were no significant changes with e′ lateral and E/e′, respectively. Thus far, HIIT has been shown to improve e′ in post‐AMI heart failure patients only.^36^ On the other hand, GLS rate showed a slight improvement only in the usual care group in our study. Similarly, the most important parameter in the prediction of ventricular remodeling, GLS, tended to improve over time, but more pronounced in the usual care group.^6^ A recent explorative non‐randomized study found no beneficial effects on left ventricular dimension and function in post‐AMI patients (including GLS and LV twist).^37^ In a comparable but larger cohort of 200 CHD patients without heart failure comparing HIIT and MICET there was no reverse cardiac remodeling over time, regardless of training modality.^38^ Of note, after completion of the program both patient groups in our study reached normal GLS values compared to those reported in healthy individuals.^39^ Moreover, radial strain showed a significant training effect with an improvement only in the HIIT group. Being aware of the technical limitations regarding radial strain, this is the first study to show an improvement in radial strain after HIIT in post‐AMI patients.^40^ This contrasts with studies that report a decrease of LV systolic (including strain analysis) and diastolic functional parameters.^41^, ^42^ However, these findings in healthy subjects and athletes seem to be transient and particularly after prolonged and strenuous exercise and are discussed controversial.

### 4.3 The impact of HIIT on cardiac biomarkers

Based on the CPET and echocardiographic findings in our study, the statement that no patient in our study developed heart failure within the first months of a first AMI is of utmost importance. This is confirmed in normal NT‐pro BNP levels in our patient cohort. On the one hand this mirrors optimized treating strategies in recent decades, but it also explains that only insignificant changes in cardiac biomarkers (particularly GRK2 expression) were detected in this first exploration of CHD patients who recently suffered an AMI undergoing HIIT.

### 4.4 Limitations

Our findings have to be interpreted in the context of numerous limitations. First of all, the sample size in the present pilot study was small with inclusion of predominantly male patients at a single tertiary institution. The initial power calculation to randomize 10 patients to each arm (20 total) calculated to have 80% power to demonstrate a significant reduction of GRK2 expression with HIIT in this population was based on a prospective exercise training study in patients with chronic heart failure.^13^ Moreover, data of one patient in the HIIT group were not available for GRK2 expression. Only eight patients in the HIIT group underwent an echocardiographic examination before and after completion of the training intervention. For the most relevant advanced echocardiographic parameters (ie, GLS) there were no data of at least one more patient in the whole cohort due to insufficient tracing and missing data.

In summary, the finding that no patient developed heart failure in our study may emphasize improved treating strategies over the last decades in this population (ie, revascularization, medication) on one hand. On the other hand, the fact that no adverse event occurred during the study together with the reported findings may indicate that HIIT is safe in this specific population. Future studies may‐be applied to a sicker cohort (ie, patients with confirmed LV dysfunction post‐AMI).

## 5 CONCLUSIONS

In patients with a recent AMI without LV dysfunction, HIIT leads to significant improvements regarding prognostic CPET parameters ($\dot{V}O_{2\text{peak}}$, OUES) compared to a usual care group. HIIT overall exhibited a more favorable cardiac remodeling with regard to echocardiographic parameters of LV function.

## CONFLICT OF INTEREST

The authors declare no potential conflict of interests.

## Supporting information


**Appendix S1**: Supplementary Material
**S1: Exclusion criteria:** Patients with a history of coronary bypass surgery, incomplete revascularisation (complete revascularization was defined as no residual major epicardial coronary artery stenosis ≥70% and no residual left main coronary artery stenosis ≥40%), myocardial necrosis in the absence of a significant flow limiting coronary artery stenosis or thrombosis, and non‐ischemic cardiomyopathy and significant valvular heart disease were excluded. NYHA class III ‐ IV symptoms, severe left ventricular dysfunction (ejection fraction ≤30%), and actively decompensated heart failure with orthopnea or paroxysmal nocturnal dyspnea were other criteria for study exclusion.
**S2: Study design and measurement:** All patients were evaluated by a cardiologist. Baseline clinical assessment included data on personal medical history including event details, cardiovascular risk factor profile, and physical examination. Anthropometric measurements included height (cm), weight (kg), body mass index, waist circumference, and body composition analysis (bio impedance, Tanita, model BC418). Blood analysis was obtained by venipuncture in the antecubital vein. During the second visit, patients underwent a maximal cardiopulmonary exercise test (CPET) for $\;\dot{V}O_{2\text{peak}}$ measurement. Within 2 weeks of enrolment a transthoracic echocardiogram was performed in the echo lab of the Montreal Heart Institute. Following baseline testing, a randomization calculator (http://randomization.com) to randomize patients 1:1 to either the HIIT or the usual care group was used.
**S3: Maximal cardiopulmonary exercise testing (CPET):** The exercise protocol started with a 3‐minute warm up phase at 20 W initial work loads. After the warm‐up phase, work load was set at 35 W followed by an increase of 15 W increments per min until exhaustion at a pedaling speed >60 rpm. The following recovery phase consisted of 2 minutes of active recovery at 20 W at pedaling speed between 50 and 60 rpm, followed by 3 minutes of passive recovery. Gas exchange parameters were continuously measured at rest, during exercise, and during recovery using a metabolic system (Oxycon Pro, CareFusion, Jaeger, Germany) as recently published. (21, 22) There was continuous monitoring of blood pressure and ECG (Marquette, case 12, St. Louis, Missouri) throughout the test. Oxygen uptake efficiency slope (OUES), ventilatory efficiency ($\;{\dot{V}}_{E}$/$\;{\dot{V}}_{\mathrm{CO}2}$) slope and Δ$\;{\dot{V}}_{\mathrm{CO}2}$/WR slope were calculated according to the recent recommendations. (28)
**S4: G protein‐coupled receptor kinase 2 evaluation:** Lymphocytes were isolated from the blood samples, and then treated with a RIPA lysis buffer. To detect GRK2 expression, 200 μg of lymphocytes‐lysate protein were resolved by electrophoresis on 10% SDS‐PAGE gels and transferred to nitrocellulose. After blocking, membranes were incubated overnight at 4oC with a mouse anti‐GRK2 antibody (dilution 1:5000; R&D Systems, #MAB43391), then washed three times with TBST and re‐incubated with a rabbit anti‐mouse horseradish peroxidase‐conjugated secondary antibody (dilution 1:5000; Abcam, #ab2089B) for 1 h30 at room temperature. Immunoreactive bands were revealed with Enhanced Chemiluminescence Substrate using BioMax BML Kodak films. The protein loading was normalized to GADH immunoreactivity (dilution 1:20000, Abcam, #ab8245). The intensity of the bands was quantified using Quantity One 1‐D Analysis Software (Bio‐Rad). The ratio GRK2/GAPDH expression was normalized in each gel to 100% in the baseline group of patients, in order to compare the changes in GRK2 pre‐ *vs* post‐training and to compare data obtained from different gels.
**S5:** **Maximal cardiopulmonary exercise test (CPET) parameters (description of the results):** There was a significant improvement in $\dot{V}O_{2\text{peak}}$ (indexed for lean body mass) with exercise training in the HIIT group but not in the usual care group (significant group x time interaction; *P* = 0.012 [g = 1.29] with +3.1 ± 2.4 mL/min/kg; *P* < 0.001 in the HIIT and + 0.1 ± 2.3 mL/min/kg; *P* > 0.05 in the usual care group, respectively). Significant group x time interaction was observed for predicted $\dot{V}O_{2\text{peak}}$ (*P* = 0.026; g = 1.12). Patients in the HIIT group improved from 93 ± 27% to 101 ± 30% of the predicted $\dot{V}O_{2\text{peak}}$ (*P* = 0.008) after training whereas in the usual care group they reached 91 ± 26% and 90 ± 25% of the predicted $\dot{V}O_{2\text{peak}}$ (*P* > 0.05). Significant group x time interaction was also observed for OUES (*P* = 0.032; g = 1.08). There was an improvement only in the HIIT group (from 1619 ± 409 to 1830 ± 481, *P* = 0.004, vs 1832 ± 399 to 1838 ± 507, *P* > 0.05 in the usual care group). Despite a non‐significant group x time interaction for O_2_ pulse (*P* = 0.110; g = 0.77) and $\dot{V}O_{2}$ at VT (*P* = 0.256; g = 0.15), these both parameters improved only in the HIIT group (*P* = 0.011 and *P* = 0.023 respectively). There was a significant overall training or time effect for the improvement of peak work load (from 120 ± 46 to 132 ± 50watts in the HIIT, *P* = 0.009 and from 127 ± 40 to 136 ± 44watts in the usual care group, *P* = 0.042).
**S6:** **Echocardiographic parameters (description of the results):** Despite a non‐significant group x time interaction for radial strain (*P* = 0.450; g = 0.40), high intensity interval training resulted in significant improvements (from 28.8 ± 9.7% pre‐ to 41.6 ± 13.3% post‐training; *P* = 0.040) while no significant change in the usual care group (from 24.5 ± 6.1% pre‐ to 31.5 ± 12.2% post‐training; *P* > 0.05). Pulsed‐wave tissue Doppler imaging (TDI) derived peak early diastolic septal mitral annulus velocity (e') showed also a non‐significant group x time interaction (*P* = 0.310; g = 0.50). However, significant increase in the HIIT group was observed (from 7.3 ± 1.2 cm/s to 8.8 ± 1.4 cm/s; *P* = 0.032) while no significant change in the usual care group (from 8.3 ± 1.6 cm/s to 8.9 ± 2.2 cm/s; *P* > 0.05). A non‐significant group x time interaction was found for global longitudinal strain rate (GLSR) (*P* = 0.616; g = 0.06) and a slight improved over time was observed in the usual care group (change [post‐pre] = −0.11 ± 0.28 second^−1^; *P* > 0.05 in the HIIT and − 0.13 ± 0.21 second^−1^; *P* = 0.042 in the usual care group). There were no significant differences with regard to global longitudinal strain, circumferential strain, left ventricular geometry (ie, left ventricular mass, end‐diastolic and end‐systolic volumes, all indexed by body surface area) and other LV diastolic functional parameters (ie, E/A ratio, E/e'; *P* > 0.05 for all reported parameters)
