# Supplementary material for: The impact of high‐intensity interval training on ventricular remodeling in patients with a recent acute myocardial infarction—A randomized training intervention pilot study
Source: Clin Cardiol. 2019 Oct 10;42(12):1222–31. doi: 10.1002/clc.23277 (PMC6906981; doi:10.1002/clc.23277)
Supplement: Supplementary file 1 — Appendix S1: Supplementary Material S1: Exclusion criteria: Patients with a history of coronary bypass surgery, incomplete revascularisation (complete revascularization was defined as no residual major epicardial coronary artery stenosis ≥70% and no residual left main coronary artery stenosis ≥40%), myocardial necrosis in the absence of a significant flow limiting coronary artery stenosis or thrombosis, and non‐ischemic cardiomyopathy and significant valvular heart disease were excluded. NYHA class III ‐ IV symptoms, severe left ventricular dysfunction (ejection fraction ≤30%), and actively decompensated heart failure with orthopnea or paroxysmal nocturnal dyspnea were other criteria for study exclusion. S2: Study design and measurement: All patients were evaluated by a cardiologist. Baseline clinical assessment included data on personal medical history including event details, cardiovascular risk factor profile, and physical examination. Anthropometric measurements included height (cm), weight (kg), body mass index, waist circumference, and body composition analysis (bio impedance, Tanita, model BC418). Blood analysis was obtained by venipuncture in the antecubital vein. During the second visit, patients underwent a maximal cardiopulmonary exercise test (CPET) for V˙O2peak measurement. Within 2 weeks of enrolment a transthoracic echocardiogram was performed in the echo lab of the Montreal Heart Institute. Following baseline testing, a randomization calculator (http://randomization.com) to randomize patients 1:1 to either the HIIT or the usual care group was used. S3: Maximal cardiopulmonary exercise testing (CPET): The exercise protocol started with a 3‐minute warm up phase at 20 W initial work loads. After the warm‐up phase, work load was set at 35 W followed by an increase of 15 W increments per min until exhaustion at a pedaling speed >60 rpm. The following recovery phase consisted of 2 minutes of active recovery at 20 W at pedaling speed between 50 and 60 rpm, [file CLC-42-1222-s001.docx]

**SUPPLEMENTAL TEXT**

**S1: Exclusion criteria**

Patients with a history of coronary bypass surgery, incomplete revascularisation (complete revascularization was defined as no residual major epicardial coronary artery stenosis ≥ 70% and no residual left main coronary artery stenosis ≥ 40%), myocardial necrosis in the absence of a significant flow limiting coronary artery stenosis or thrombosis, and non-ischemic cardiomyopathy and significant valvular heart disease were excluded. NYHA class III - IV symptoms, severe left ventricular dysfunction (ejection fraction ≤ 30%), and actively decompensated heart failure with orthopnea or paroxysmal nocturnal dyspnea were other criteria for study exclusion.

**S2: Study design and measurement**

All patients were evaluated by a cardiologist. Baseline clinical assessment included data on personal medical history including event details, cardiovascular risk factor profile, and physical examination. Anthropometric measurements included height (cm), weight (kg), body mass index, waist circumference, and body composition analysis (bio impedance, Tanita, model BC418). Blood analysis was obtained by venipuncture in the antecubital vein. During the second visit, patients underwent a maximal cardiopulmonary exercise test (CPET) for$\dot{V}$O_2peak_ measurement. Within 2 weeks of enrolment a transthoracic echocardiogram was performed in the echo lab of the Montreal Heart Institute. Following baseline testing, a randomization calculator (randomization.com) to randomize patients 1:1 to either the HIIT or the usual care group was used.

**S3: Maximal cardiopulmonary exercise testing (CPET)**

The exercise protocol started with a 3-minute warm up phase at 20 W initial work loads. After the warm-up phase, work load was set at 35 W followed by an increase of 15 Watt increments per min until exhaustion at a pedalling speed > 60 rpm. The following recovery phase consisted of 2 minutes of active recovery at 20 W at pedalling speed between 50 and 60 rpm, followed by 3 minutes of passive recovery. Gas exchange parameters were continuously measured at rest, during exercise, and during recovery using a metabolic system (Oxycon Pro, CareFusion, Jaeger, Germany) as recently published (21,22). There was continuous monitoring of blood pressure and ECG (Marquette, case 12, St. Louis, Missouri) throughout the test. Oxygen uptake efficiency slope (OUES), ventilatory efficiency ($\dot{V}$E/$\dot{V}$CO2) slope and ∆$\dot{V}$O_2_/WR slope were calculated according to the recent recommendations (28).

**S4: G protein–coupled receptor kinase 2 evaluation**

Lymphocytes were isolated from the blood samples, and then treated with a RIPA lysis buffer. To detect GRK2 expression, 200 µg of lymphocytes-lysate protein were resolved by electrophoresis on 10% SDS-PAGE gels and transferred to nitrocellulose. After blocking, membranes were incubated overnight at 4oC with a mouse anti-GRK2 antibody (dilution 1:5,000; R&D Systems, #MAB43391), then washed three times with TBST and re-incubated with a rabbit anti-mouse horseradish peroxidase-conjugated secondary antibody (dilution 1:5,000; Abcam, #ab2089B) for 1h30 at room temperature. Immunoreactive bands were revealed with Enhanced Chemiluminescence Substrate using BioMax BML Kodak films. The protein loading was normalized to GADH immunoreactivity (dilution 1: 20,000, Abcam, #ab8245). The intensity of the bands was quantified using Quantity One 1-D Analysis Software (Bio-Rad). The ratio GRK2/GAPDH expression was normalized in each gel to 100% in the baseline group of patients, in order to compare the changes in GRK2 pre- *versus* post-training and to compare data obtained from different gels.

S5: **Maximal cardiopulmonary exercise test (CPET) parameters (description of the results)**

There was a significant improvement in $\dot{V}$O_2peak_ (indexed for lean body mass) with exercise training in the HIIT group but not in the usual care group (significant group x time interaction; p=0.012 (g=1.29) with +3.1 ± 2.4ml/min/kg; p < 0.001 in the HIIT and +0.1 ± 2.3ml/min/kg; p>0.05 in the usual care group, respectively). Significant group x time interaction was observed for predicted $\dot{V}$O_2peak_ (p=0.026; g=1.12). Patients in the HIIT group improved from 93 ± 27% to 101 ± 30% of the predicted $\dot{V}$O_2peak_ (p=0.008) after training whereas in the usual care group they reached 91 ± 26% and 90 ± 25% of the predicted $\dot{V}$O_2peak_ (p > 0.05). Significant group x time interaction was also observed for OUES (p=0.032; g=1.08). There was an improvement only in the HIIT group (from 1619 ± 409 to 1830 ± 481, p=0.004, vs. 1832 ± 399 to 1838 ± 507, p > 0.05 in the usual care group). Despite a non-significant group x time interaction for O_2_ pulse (p=0.110; g=0.77) and $\dot{V}$O_2_ at VT (p=0.256; g=0.15), these both parameters improved only in the HIIT group (p=0.011 and p=0.023 respectively). There was a significant overall training or time effect for the improvement of peak work load (from 120 ± 46 to 132 ± 50watts in the HIIT, p=0.009 and from 127 ± 40 to 136 ± 44watts in the usual care group, p=0.042).

S6: **Echocardiographic parameters (description of the results)**

Despite a non-significant group x time interaction for radial strain (p=0.450; g=0.40), high intensity interval training resulted in significant improvements (from 28.8 ± 9.7% pre- to 41.6 ± 13.3% post-training; p=0.040) while no significant change in the usual care group (from 24.5 ± 6.1% pre- to 31.5 ± 12.2% post-training; p > 0.05). Pulsed-wave tissue Doppler imaging (TDI) derived peak early diastolic septal mitral annulus velocity (e’) showed also a non-significant group x time interaction (p=0.310; g=0.50). However, significant increase in the HIIT group was observed (from 7.3 ± 1.2cm/s to 8.8 ± 1.4cm/s; p=0.032) while no significant change in the usual care group (from 8.3 ± 1.6cm/s to 8.9 ± 2.2cm/s; p > 0.05). A non-significant group x time interaction was found for global longitudinal strain rate (GLSR) (p=0.616; g=0.06) and a slight improved over time was observed in the usual care group (change (post-pre) = -0.11 ± 0.28 s^-1^; p > 0.05 in the HIIT and -0.13 ± 0.21 s^-1^; p=0.042 in the usual care group). There were no significant differences with regard to global longitudinal strain, circumferential strain, left ventricular geometry (i.e. left ventricular mass, end-diastolic and end-systolic volumes, all indexed by body surface area) and other LV diastolic functional parameters (i.e. E/A ratio, E/e’; p > 0.05 for all reported parameters).
